# Paclitaxel–ifosfamide–carboplatin combination chemotherapy regimen in advanced uterine and adnexal malignant mixed Mullerian tumours

**DOI:** 10.1038/bjc.2011.316

**Published:** 2011-08-16

**Authors:** C Kosmas, G Vorgias, G Tsakonas, P Politis, T Daladimos, E Panagiotidi, T Papachrysanthou, D Moschovis, N Kalinoglou, N Tsavaris, A Karabelis, N Mylonakis

**Affiliations:** 1 Second Division of Medical Oncology, Department of Medicine, ‘Metaxa’ Cancer Hospital, Piraeus, Greece; 2 Department of Gynecology, ‘Metaxa’ Cancer Hospital, Piraeus, Greece; 3 Medical Oncology Unit, Department of Pathophysiology, Athens University School of Medicine, ‘Laikon’ General Hospital, Athens, Greece

**Keywords:** paclitaxel, ifosfamide, carboplatin, malignant mixed Mullerian tumours

## Abstract

**Background:**

Malignant mixed Mullerian tumours (MMMTs) of the uterus and adnexa represent aggressive gynaecologic malignancies with a high rate of loco-regional and distant failure. For that reason, we evaluated the paclitaxel–ifosfamide–carboplatin (TICb) combination in patients with advanced MMMTs.

**Methods:**

Female patients with advanced MMMTs, WHO-PS 0–2, no prior chemotherapy for systemic disease, unimpaired haemopoietic and organ function were eligible. Chemotherapy was administered at the following doses; paclitaxel: 175 mg m^–2^ on day 1, ifosfamide: 2.0g m^–2^ day^–1^ – days 1 and 2, and carboplatin at a target area under the curve 5 on day 2, with prophylactic G-CSF from day 3.

**Results:**

Forty patients of a median age 61 (45–72) years, performance status 0–2 with advanced MMMTs of the uterus (*n*=34), tubes (*n*=2) or ovary (*n*=4) have entered and all were evaluable for response and toxicity. Responses were as follows: 27 out of 40 (67.5%) evaluable patients responded, with 11 complete responses and 16 partial responses, while 10 had stable disease, and 3 developed progressive disease. The median response duration was 9 months (range, 4–40 months), median progression-free survival 13 months (range, 3–42 months), while median overall survival 18 months (range, 4–48 months). Grade 3/4 neutropenia was recorded in 22 out of 40 (55%) – with 13 developing grade 4 (⩽7 days) and 7 out of 40 (17.5%) of patients at least one episode of febrile neutropenia.

**Conclusion:**

In this study, it appears that the TICb combination, yielded important activity with manageable toxicity in females with advanced MMMTs warranting further randomised comparison with current standard regimens.

Malignant mixed Mullerian tumours (MMMTs) or carcinosarcomas of the uterus and adnexa represent aggressive gynaecologic malignancies with a high rate of loco-regional and distant failure. Previously untreated disseminated carcinosarcomas (MMMTs) of the uterus carry a worse prognosis when compared with classical uterine adenocarcinoma, and are associated with shorter survival (Hensley, 2006). Single-agent chemotherapy response rates (RRs) in previous Gynaecologic Oncology Group (GOG) studies were; ifosfamide 32% (Sutton *et al*, 1989), cisplatin 19% (Thigpen *et al*, 1991) as first-line agents and paclitaxel 18% at second-line (Curtin *et al*, 2001). In contrast to leiomyosarcoma and undifferentiated endometrial sarcoma, doxorubicin has been only minimally active in MMMTs with a 10% RR (Omura *et al*, 1983).

Combination of ifosfamide and cisplatin (GOG study) has yielded better RRs than single-agent ifosfamide, however, without an advantage in overall survival (OS) and at the expense of increased toxicity (Sutton *et al*, 2000). A recent GOG-161 study comparing ifosfamide+paclitaxel *vs* single-agent ifosfamide yielded significantly better RRs (45 *vs* 29%), prolongation in median progression-free survival (PFS) (5.8 *vs* 3.6 months) and OS (13.5 *vs* 8.4 months) (Homesley *et al*, 2007). With single-agent activity documented for both paclitaxel and cisplatin, there is considerable interest in treating advanced MMMTs with paclitaxel plus carboplatin, as it has been a well-tolerated regimen, with preliminary reports of high RRs.

Given the documented superiority of the paclitaxel+ifosfamide combination over ifosfamide, as well as the encouraging activity and tolerability of the paclitaxel+carboplatin regimen, in this study, we sought to evaluate the paclitaxel–ifosfamide–carboplatin (TICb) combination in patients with advanced MMMTs.

## Patients and methods

### Patient selection

Consecutive patients with histologically confirmed advanced MMMTs of the uterus or adnexa and no prior chemotherapy were candidates for treatment with the TICb combination chemotherapy regimen. Eligibility included: (i) patients aged between 18–75 years with histologically confirmed MMMTs not potentially curable by other local measures such as surgery or radiotherapy, (ii) WHO performance status ⩽2, (iii) life expectancy ⩾3 months, (iv) adequate haemopoietic (ANC>1.500 *μ*l^–1^, PLT>100.000 *μ*l^–1^), liver (bilirubin<1.5 mg dl^–1^, AST/ALT<2 × upper normal limit (nl), unless caused by tumour and serum albumin>3.0 g dl^–1^) and renal function (BUN<1.5 nl; nl=23; and creatinine<1.5 nl; nl=1.5 mg dl^–1^ in our laboratory or creatinine clearance (CrCl)>60 ml min^–1^), (v) absence of active coronary artery disease (in the form of unstable angina or myocardial infarction over the last 12 months), unstable diabetes mellitus or peripheral neuropathy ⩾grade 2 by the WHO criteria, (vi) no prior irradiation to areas encompassing > 20% of marrow-bearing bone, apart from pelvic radiotherapy, (vii) presence of bi-dimensionally measurable disease outside a previously irradiated field, unless definite evidence of progression at this site. Patients with isolated progression within the pelvis after radical surgery±radiotherapy had to demonstrate a ⩾50% increase in the sum of the products of residual lesions at least 3 months after completion of radiotherapy. Patients with brain metastases were excluded in this study. Informed consent was obtained from each patient before study entry according to Institutional policies, and the study was conducted according to Helsinki declaration and approved by the participating Institutions.

### Treatment schedule

Eligible patients were treated as follows: paclitaxel was administered at 175 mg m^–2^ over 1–3 h by i.v. infusion on day 1, after premedication consisting of dexamethasone 20 mg, dimethidene maleate (Fenistil) 4 mg and ranitidine 50 mg; all administered i.v. 1 h before paclitaxel. Ifosfamide was administered at 2.0 g m^–2^ i.v. over 1 h for 2 days (days 1, 2: total dose 4.0 g m^–2^) together with mesna uroprotection, 40% of the ifosfamide dose, given i.v. together with ifosfamide infusion, and at 3 and 6 h thereafter. Carboplatin was administered at an area under the curve (AUC)=5 i.v. over 30 min on day 2 after ifosfamide. The dose of carboplatin was based on CrCl calculated according to the modified Cockcroft–Gault formula (CrCl=(140 – age) × (actual weight)/(72 × serum creatinine in mg dl^–1^)) × 0.85 (for females) (intended ages 18–110 and serum creatinine values 0.6–7 mg dl^–1^) at an area under the concentration × time curve (AUC)=5 (according to Calvert formula; carboplatin dose (mg)=(CrCl+25) × AUC=5). The chemotherapy schedule (TICb) was recycled every 21 days. A majority of the patients were treated as in-patients, however, certain patients were treated in the outpatient setting.

### Supportive care

Standard antiemetic medication included ondansetron 24 mg or granisetron 3 mg i.v. 1 h before chemotherapy, at 12 h 8 mg i.v./p.o. or 3 mg i.v./1 mg p.o., respectively, on days 1, 2. Dexamethasone 20 mg i.v. was administered 1 h before chemotherapy (day 1 as paclitaxel pre-medication as well) on days 1, 2 and post-chemotherapy 4 mg t.i.d. p.o. on days 3–5. Patients enrolled over the last 2 years additionally received aprepitant (Emend) 125 mg i.v. on day 1 and 80 mg p.o. on days 2 and 3 for prevention of delayed emesis.

Haemopoietic growth factors included G-CSF 5 μg kg^–1^ s.c. (filgrastim) from day 4 – until WBC⩾5.000 μl^–1^, and recombinant human erythropoietin (rh-Epo) 30 000 IU Epoetin-beta or 40 000 IU Epoetin-alpha × 1 per week (not on the days of chemotherapy) whenever the haemoglobin (Hb) value dropped ⩽10.5 g dl^–1^ and continued until Hb ⩾12 g dl^–1^.

### Dose modifications for toxicity

The prerequisites for dose modifications were set as follows: (i) any episode of grade 4 neutropenia of >7 days duration, (ii) any episode of febrile grade 4 neutropenia, (iii) any episode of grade 4 thrombocytopenia and (iv) any non-haematologic grade 3 or 4 toxicity excluding nausea and vomiting, musculoskeletal and arthritic pain (myalgia/arthralgia syndrome) and alopecia.

The following guidelines were applied with respect to dose reductions for toxicity: (i) for neutropenia and thrombocytopenia meeting the aforementioned criteria, drug doses were reduced by 20% in subsequent cycles and if toxicity reappeared after a total of 40% reduction from the starting dose in consecutive cycles treatment was stopped, however, the patient was evaluable for toxicity and response, (ii) for ⩾grade 3 mucositis the doses of paclitaxel and ifosfamide were reduced by 20% in subsequent cycles, (iii) for neuropathy ⩾grade 3 treatment was interrupted, (iv) for renal toxicity ⩾grade 3 (serum creatinine elevations >3 × normal) treatment was withheld until recovery (serum creatinine <1.8 mg dl^–1^) with carboplatin and ifosfamide administered with more post-hydration and hospitalisation in subsequent cycles. If the GFR dropped to <50 ml min^–1^, ifosfamide was omitted in subsequent cycles, and carboplatin dose adjusted to GFR according to Calvert's formula, and (v) for ⩾grade 3 CNS toxicity (ifosfamide encephalopathy) the dose of ifosfamide was reduced by 20% and more hydration with bicarbonates was anticipated in subsequent cycles. In the case that encephalopathy reappeared, ifosfamide was omitted from subsequent cycles. In the case that blood counts had not recovered to ANC ⩾1.500 *μ*l^–1^ and PLT ⩾100 000 *μ*l^–1^ on the day of therapy, treatment was withheld until recovery, and after a maximum delay of 2 weeks no further therapy was administered.

### Pretreatment, follow-up studies and response evaluation

Tumour measurements were performed by physical examination and the specific radiological test that documented measurable disease before treatment. Before the first chemotherapy cycle a detailed clinical and gynaecologic (pelvic) examination followed by CT scans of the chest/abdomen/pelvis and radionuclide bone scintigraphy were carried out in all patients. CT scans of the brain were carried-out in the case of suspected brain metastases. Clinical examination, full blood counts, biochemical tests, appropriate serum tumour marker measurements and a chest X-ray were carried-out before each cycle of therapy. Blood counts were checked weekly after each cycle (days 8 and 15) or more frequently in the case of grade 3/4 haematologic toxicity. Evaluation of response was performed every three cycles of therapy. Patients experiencing toxic death despite objective responses at measurable sites would be categorised as treatment failures. Definitions of response; namely complete remission (CR), partial remission (PR), stable disease (SD) and progressive disease (PD), were carried-out according to WHO criteria as the study initiated in 2001 (Miller *et al*, 1981).

### Statistical methods

Patients who received at least two cycles of treatment were evaluable for response unless they had definite evidence of progression after the first cycle were categorised as having PD, and patients who received at least one cycle of treatment were evaluable for toxicity. Response duration was measured from the day of its initial documentation until PD; PFS was calculated from the date of treatment initiation until evidence of PD; OS was measured from the day of entry until last follow-up or death. The 95% CI for RRs were calculated from the binomial distribution (Cox, 1970). Actuarial survival was estimated by the product-limit method of Kaplan and Meier, 1959. Patients fulfilling the eligibility criteria were entered and evaluated consecutively in a prospective manner. The study followed the design of phase II studies, with RR as the main end point. According to Simon's two-stage design (1989), with a sample size of *n*=40, the study has 80% power to accept the hypothesis that the true RR is >50%, while *P*<0.05 to reject the hypothesis that RR is <30%. At the first stage, if <5 responses occurred out of the initial 16 patients, the study would conclude that the anticipated RR was <30% and terminate, with a power >90%.

## Results

### Patients’ characteristics

Between July 2001 and August 2009, 40 patients with relapsed/metastatic MMMTs were treated with the TICb chemotherapy regimen. Final data analysis was carried-out in September 2010, after all patients entered had completed the planned six cycles of chemotherapy or interrupted treatment as a result of disease progression or unacceptable toxicity. Patient characteristics and demographics are provided in Table 1. Median age was 58 years (range, 45–72 years), and 90% had a WHO-PS 0 or 1, whereas 10% had a WHO-PS=2. Distribution of tumour primaries was as follows; uterus 85%, tubes 5% and ovaries 10%. Overall, 55% of patients had surgery and 30% had surgery followed by pelvic radiotherapy. Surgery consisted of total abdominal hysterectomy, salphingo-ophorectomy and omentectomy. Histologies included homologous sarcoma component in 65% and heterologous sarcoma component in 35% of cases.

### Response to treatment and survival

**Figure 1:**
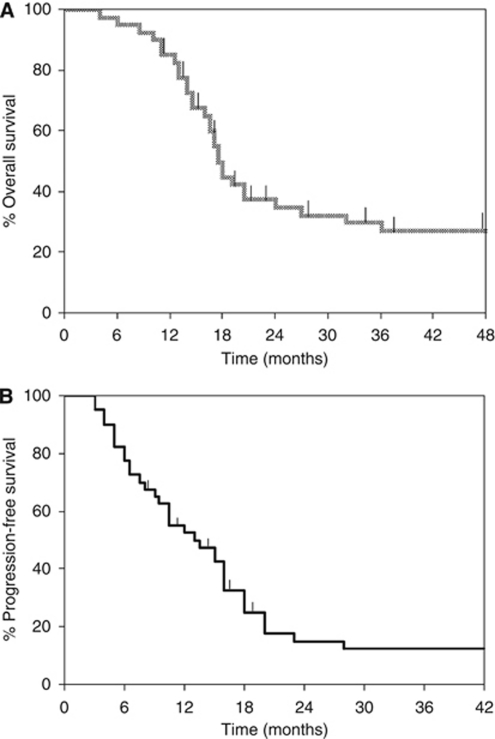
Kaplan–Meier data on (**A**) OS and (**B**) PFS. Tick marks indicate censored observations.

Response to TICb chemotherapy were as follows: 27 out of 40 evaluable patients responded for an overall RR=67.5% (95% CI, 53–82%); with 11 CR; 27.5% (95% CI, 13.7–41.3%) and 16 PR; 40% (95% CI, 24.8–55.2%), while 10 had SD; 25% (95% CI, 26.6–53.4%), and 3 developed PD; 7.5% (95% CI, 0–15.7%). More specifically, 4 out of 6 (67%) patients with ovarian/tubal and 23 out of 34 (67.6%) patients with uterine MMMTs responded. The median response duration was 9 months (range, 4–40 months), median OS 18 months (range, 4–48 months) (Figure 1A) and median PFS was 13 months (range, 3–42 months) (Figure 1B).

### Compliance to treatment

A total of 224 treatment cycles (median: 6 cycles; range, 2–6, mean: 5.33 cycles) were administered. Six patients did not complete the planned six cycles as a result of PD; detected after the third cycle in three patients and after cycles 4 and 5 in another three patients. Four more patients did not complete the planned six cycles as a result of: two dose reductions in successive cycles for haematologic toxicity (as defined above) in two patients after the fourth and fifth cycle, and treatment omission for renal toxicity in two patients (after cycle 2 and 4).

### Toxicities

Haematologic and non-haematologic toxicity data for all patients enrolled are summarised in Tables 2 and 3, respectively. Haematologic toxicities (Table 2) consisted primarily of grade 3/4 neutropenia in 55% (32.5% grade 4) of patients despite prophylactic G-CSF administration, while grade 3/4 thrombocytopenia was encountered in 22.5% (7.5% grade 4) of patients. Febrile neutropenia was seen in 7 out of 40 (17.5%) of patients with 3 of them developing more than one episode. All febrile neutropenic events were managed successfully in the in-patient or outpatient setting by broad spectrum antibiotics, and there were no treatment-related deaths. rh-Epo was required by 17 (42.5%) patients and during 67 (30%) treatment cycles, while blood transfusions were administered in 6 out of 40 (15%) of patients on study.

Non-haematologic toxicities (Table 3) consisted primarily of grade 2/3 nausea-vomiting in 10 out of 15% of patients, grade 1/2 myalgia-arthralgia in 15 out of 40% and mild grade 1 mucositis in 15% with no ⩾grade 2. Grade 2 peripheral neuropathy was observed in 10% of patients and usually resolved to ⩽grade 1 in the majority.

### Dose-intensity analysis

The administered median dose-intensities for each drug of the TICb combination were as follows: for paclitaxel 52.0 mg m^–2^ per week (range: 48.3–58.3) or 89.2% (range: 82.2–100%) of the planned dose, for ifosfamide 1.27 g m^–2^ per week (range: 1.2–1.33) or 95% (range: 90–100%) of the planned dose and carboplatin AUC=1.67 per week (range: 1.33–1.67) or 100% (range: 80–100%) of the planned dose. Therefore, patients received >85% of the planned dose-intensity for all cytotoxic drugs in the regimen.

## Discussion

Carcinosarcomas of the female genital system, also termed MMMTs, represent aggressive malignancies often diagnosed at advanced stages (III or IV) in the majority of cases. Despite diagnosis at earlier stages in some cases, prognosis is still grim as a result of early local and/or disseminated recurrences. A recent GOG randomised study, evaluating ifosfamide+cisplatin ( × 3 cycles) *vs* whole abdominal–pelvic irradiation in the adjuvant setting, demonstrated improved survival figures with chemotherapy, however, half of the patients died of recurrent disease despite early disease stages (I or II) and surgically debulked more advanced stages (Sutton *et al*, 2005).

Ifosfamide and cisplatin have traditionally represented the most active agents in MMMTs and their combination formed a standard for future comparisons. An initial GOG randomised study in advanced stages compared the combination of ifosfamide+cisplatin *vs* single-agent ifosfamide and despite improved RRs and median PFS, survival prolongation did not reach significance (*P*=0.07), at the expense of more severe haematologic and renal toxicity for the combination (Sutton *et al*, 2000). However, the initially selected doses of ifosfamide 1.5g m^–2^ day^–1^ and cisplatin 20 mg m^–2^ day^–1^ both × 5 days appeared non-feasible as a result of severe haematologic, renal and CNS toxicities leading to an unacceptably high toxic death rate in the initial patients treated. Despite a 20% dose reduction for both drugs in subsequent patients, there were still concerns of significant toxicity, particularly haematologic and CNS, leading investigators to conclude that, addition of ifosfamide to cisplatin despite offering a small improvement in PFS over ifosfamide alone, the added toxicity may not justify the use of this combination (Sutton *et al*, 2000). Therefore, based on this experience and the encouraging single-agent activity of paclitaxel in advanced disease (Curtin *et al*, 2001), a subsequent GOG-161 study omitted cisplatin and compared single-agent ifosfamide *vs* paclitaxel+ifosfamide, yielding superiority of the combination in terms of RR, PFS and OS (Homesley *et al*, 2007).

Over the last 15 years, the combination of paclitaxel and carboplatin has gained much popularity in a variety of advanced solid tumours and gynaecologic malignancies given its consistent activity, acceptable toxicity profile and ease of administration in the outpatient setting. Anecdotal reports and small phase II studies have demonstrated encouraging activity of the paclitaxel+carboplatin combination in uterine and adnexal MMMTs (Sit *et al*, 2000; Duska *et al*, 2002; Toyoshima *et al*, 2004; Kawaguchi *et al*, 2008).

Preclinical data have demonstrated that paclitaxel intensifies the cell-killing effects of chemically induced DNA damage by alkylating agents and cisplatin, provided that paclitaxel precedes these agents (Parker *et al*, 1993; Liebmann *et al*, 2004). In the clinical setting, paclitaxel has shown enhanced activity and possibly synergistic effects when combined with alkylating agents; cyclophosphamide and ifosfamide (Bunnell *et al*, 1998), or cisplatin (Rowinsky *et al*, 1993). The proposed mechanisms of *in vitro* and *in vivo* synergism have been discussed previously (Lind *et al*, 1989; Reed *et al*, 1995; Kosmas *et al*, 2009). On the basis of the above data, the established activity of ifosfamide+ciplatin and the favourable preliminary results of the paclitaxel+carbolatin combination, in 2001, we elected to evaluate the combination of TICb in advanced MMMTs. To our knowledge, this study represents the largest single-institution prospective series evaluating a three-drug regimen (TICb) in relapsed/metastatic MMMTs. The TICb regimen, as applied in this study, yielded efficacy and survival figures that compare favourably with those obtained in other GOG studies with ifosfamide+cisplatin (Sutton *et al*, 2000), paclitaxel+ifosfamide (Homesley *et al*, 2007) and a recently completed phase II study with paclitaxel+carboplatin (Powell *et al*, 2010). This latter study demonstrated a 54% ORR, 7.6 months PFS and 14.7 months OS *vs* 67.5%, 13 and 18 months, in this study, respectively. However, in the GOG study RRs were reported on the actual number of 46 patients that were finally treated-evaluable out of the initially 55 enrolled and not on an intent-to-treat basis (Powell *et al*, 2010). However, it should be noted that the GOG study included elderly patients, with about 40% of their subjects being 70–80 years old, as well as 34.8% of their patients had recurrent disease after loco-regional treatment *vs* 20% in our study population. Moreover, our study included six patients with ovarian/tubal MMMTs, while others had only uterine MMMTs. Similarly, with the limitations of inter-study comparisons in mind, the TICb combination compares favourably with other paclitaxel–carboplatin studies in advanced MMMTs that yielded RRs of 55–80% (Toyoshima *et al*, 2004; Ramondetta *et al*, 2007; Hoskins *et al*, 2008). Only two of the above studies were prospective phase II. In the British Columbia study, 32 out of 40 enrolled patients were evaluable, and ORRs were 60% for patients with newly diagnosed advanced MMMTs and 55% for patients with recurrent MMMTs (Hoskins *et al*, 2008). The second study has been reported in abstract form, and 18 out of 37 planned sample had been treated at the time of the report. As seven patients were treated in the adjuvant setting, this left a group of only 11 patients evaluable, with a documented RR of 64% (Ramondetta *et al*, 2007). In another small retrospective series, six patients were treated with the paclitaxel+carboplatin combination and four out of five (80%) evaluable patients responded (Toyoshima *et al*, 2004). Results between studies may vary as a consequence of differences in the PS of enrolled patients, inclusion of different proportions of relapsed patients exposed to prior pelvic radiotherapy, and drug doses.

Despite favourable results with the TICb combination in the present prospective phase II study, these were achieved at the expense of significant haematologic toxicity with an 18% incidence of febrile neutropenia despite routine prophylactic G-CSF administration. At present, administration and monitoring of TICb appears to be limited at experienced Oncology centres rather than a multi-institutional setting. However, the majority of the febrile neutropenic episodes were managed uneventfully in the outpatient setting with very rare hospital admissions. Moreover, no other severe toxicities were recorded and no treatment-related deaths were observed.

Despite improvements in RRs with the paclitaxel+carboplatin or the current TICb combinations with acceptable or manageable toxicities, the long-term prognosis of advanced MMMTs remains grim with very few patients surviving beyond 2 years. It is therefore anticipated that novel cytotoxic and biologic agents and their combinations are required to be tested in this setting. Ongoing studies in advanced/metastatic MMMTs include: (i) anti-angiogenic agents, such as sorafenib, sunitinib and VEGF-trap, (ii) kinase inhibitors; AZD0530 and BI-2536, (iii) newer cytotoxic drugs, such as temozolomide, pegylated-liposomal doxorubicin (PLD) (combined with carboplatin), trabectedin with established activity in uterine sarcomas and epithelial ovarian cancer at second-line, (iv) combinations of BSI-201, a DNA repair inhibitor, with paclitaxel+carboplatin, and (v) combination of the first-in class proteasome inhibitor bortezomib with gemcitabine (for details on all the above studies see also at; http://clinicaltrials.gov). Despite the absence of as yet available phase II data on single-agent PLD or PLD+carboplatin, a recently reported single-institution series evaluated the three-drug combination of paclitaxel–PLD–carboplatin in 29 patients with advanced uterine MMMTs yielding a 62% RR, 8.2 months median PFS and 16.4 months median OS. Toxicities were as follows; FN 10%, grade 3/4 thrombocytopenia 31%, grade 3 peripheral neuropathy 10% and palmar-plantar erythrodysesthesia 8% (Pectasides *et al*, 2008).

Our results highlight the feasibility and important activity of TICb combination in relapsed and/or metastatic MMMTs, however, at the cost of increased but manageable haematologic toxicity. Given the toxicity, cost and difficulty in administering the current three-drug regimen (TICb) in a multi-institutional setting, it is the authors’ belief that randomised phase III comparisons of TICb *vs* either paclitaxel+carboplatin or paclitaxel+ifosfamide doublets are not currently justifiable. Further refinements of the TICb combination and appropriately designed phase II studies might be conducted before these comparisons can be realised.

## Figures and Tables

**Table 1:** Patient characteristics

**Characteristic**	**No.**	**%**
Total patients	40	100
*Age (years)*		
Median (range)	58 (45–72)	
*Performance status (WHO)*		
0–1	36	90
2	4	10
		
*Tumour location*		
Uterus	34	85
Tubes	2	5
Ovary	4	10
		
*Disease extent*		
Stage III	18	45
Stage IV	14	35
Recurrent (stages I/II at Dx)	8	20
		
*Histology*		
Homologous sarcoma	26	65
Heterologous sarcoma	14	35
		
*Previous therapy*		
Surgery	22	65
Surgery+RT	12	30
None	6	15
		
*Metastatic sites*		
Pelvic mass	16	40
Pelvic/paraortic LNs		40
Peritoneal implants		45
Liver	4	10
Lung nodules	12	30
Pleural effusion	4	10
Bone	2	5

Abbreviations: Dx=diagnosis; LN=lymph node; RT=radiotherapy; WHO=World Health Organisation.

**Table 2:** Haematologic toxicities (NCI-CTC grade)

	**Grade (% of patients, all cycles)**	
**Toxicity**	**3**	**4**
Leukopenia	50	45
Neutropenia	22.5	32.5
Thrombocytopenia	15	7.5
Anaemia	37	0
		
Febrile neutropenia^a^	7/40 (17.5%)	

Abbreviation: NCI-CTC=National Cancer Institute-Common Toxicity Criteria.

a Managed successfully by broad-spectrum antibiotics in all (in three with home p.o. therapy with amoxicillin/clavulanate+ciproxin).

**Table 3:** Non-haematologic toxicities (NCI-CTC grade)

	**NCI-CTC grade (% of patients, all cycles)**				
**Toxicity**	**0**	**1**	**2**	**3**	**4**
Nausea and vomiting	65	10	10	15	
Mucositis	95	5	0	0	0
Myalgia/arthralgia	45	15	40	0	—
					
*Neurologic*					
Peripheral	70	20	10	0	0
CNS	97.5	2.5	0	0	0
Diarrhoea	75	20	5	0	—
Allergy	85	10	5	0	0
Alopecia	0	0	100	0	—
Asthenia/fatigue	82.5	10	7.5	0	—
Cardiac	100	0	0	0	0
Pulmonary	100	0	0	0	0
Renal	100	0	0	0	0
Haematuria	100	0	0	0	0

Abbreviations: CNS=central nervous system; NCI-CTC=National Cancer Institute-Common Toxicity Criteria.
